# High-Frequency Transcranial Random Noise Stimulation Enhances Perception of Facial Identity

**DOI:** 10.1093/cercor/bhv016

**Published:** 2015-02-06

**Authors:** Aleksandra Romanska, Constantin Rezlescu, Tirta Susilo, Bradley Duchaine, Michael J. Banissy

**Affiliations:** 1 Department of Psychology, Goldsmiths, University of London, London SE14 6NW, UK; 2 Department of Psychology, Harvard University, Cambridge, MA 02138, USA; 3 Department of Psychological and Brain Sciences, Dartmouth College, Hanover, NH, USA

**Keywords:** brain stimulation, face perception, facial identity, transcranial current stimulation, transcranial random noise stimulation

## Abstract

Recently, a number of studies have demonstrated the utility of transcranial current stimulation as a tool to facilitate a variety of cognitive and perceptual abilities. Few studies, though, have examined the utility of this approach for the processing of social information. Here, we conducted 2 experiments to explore whether a single session of high-frequency transcranial random noise stimulation (tRNS) targeted at lateral occipitotemporal cortices would enhance facial identity perception. In Experiment 1, participants received 20 min of active high-frequency tRNS or sham stimulation prior to completing the tasks examining facial identity perception or trustworthiness perception. Active high-frequency tRNS facilitated facial identity perception, but not trustworthiness perception. Experiment 2 assessed the spatial specificity of this effect by delivering 20 min of active high-frequency tRNS to lateral occipitotemporal cortices or sensorimotor cortices prior to participants completing the same facial identity perception task used in Experiment 1. High-frequency tRNS targeted at lateral occipitotemporal cortices enhanced performance relative to motor cortex stimulation. These findings show that high-frequency tRNS to lateral occipitotemporal cortices produces task-specific and site-specific enhancements in face perception.

## Introduction

In our daily lives, faces are a major source of social information. Faces allow us to distinguish between friends and foes, and infer others' emotional states. Face processing difficulties are present in autism and other neurodevelopmental conditions, and contribute to deficits in communication and social competence, reduced quality of life, and social isolation (Feldman and Rime 1991; Yardley et al. 2008; Kanai et al. 2012; Bate et al. 2013; Dalrymple et al. 2014). Conversely, some individuals process faces extraordinarily well (Russell et al. 2009, 2012), and such skills are valuable for law enforcement and other security organizations (White et al. 2014). Given the importance of face processing, techniques that enhance it could be valuable.

Face processing has received extensive attention in cognitive neuroscience. For example, functional magnetic resonance imaging has highlighted several neural regions that respond strongly to faces including areas in occipitotemporal cortex and frontal brain regions [e.g., see Haxby et al. (2000) and Weiner and Grill-Spector (2013) for review]. Disruptions to these regions through lesions (Dalrymple et al. 2011; Rossion 2014) and brain stimulation indicate that they play a causal role in face recognition (Pitcher et al. 2009; Jonas et al. 2014), but not in other visual classes (e.g., Pitcher et al. 2009; Kim et al. 2011; Dilks et al. 2013).

Previous studies using brain stimulation have disrupted face recognition (e.g., Pitcher et al. 2009; Dilks et al. 2013), but transcranial current stimulation (tCS; Paulus et al. 2013) is an alternative stimulation technique that may provide a means to improve face recognition. In the case of tCS, 2 electrodes are placed on the scalp, which stimulate the cerebral cortex with a weak electric current to manipulate cortical excitability. This technique has been shown to have particular utility in improving cognitive and perceptual abilities (e.g., Cohen Kadosh et al. 2010; Santiesteban et al. 2012; Tseng et al. 2012; Cappelletti et al. 2013; Ruff et al. 2013; Snowball et al. 2013; Hogeveen et al. forthcoming). In the context of face processing, it has also been used to examine dissociations between brain regions involved in facial emotion perception (e.g., Tseng et al. 2014) and functional interactions between prefrontal and occipitotemporal brain regions in the visual encoding of unfamiliar faces (e.g., Lafontaine et al. 2013). This approach, therefore, not only provides an interesting avenue to inform us about what role different brain regions play in psychological process, but also to determine potential means by which performance may be improved.

Here, we employed high-frequency transcranial random noise stimulation (tRNS) targeted at lateral occipitotemporal cortices across 2 studies to determine whether facial identity processing could be improved following a single session of high-frequency tRNS. tRNS is a relatively novel technique that involves the generation of a random level of current for every sample passed between the 2 electrodes. The effect of this type of tCS is an increase in cortical excitability under both electrodes placed on the scalp (Terney et al. 2008). In other domains (e.g., numerical cognition—Cappelletti et al. 2013; Snowball et al. 2013), tRNS has been shown to facilitate performance, but as yet it has not been used to modulate facial processing. Here, we address this by first examining whether facial identity perception could be improved in a task-specific manner following high-frequency tRNS targeted at lateral occipitotemporal cortices, and then in a second experiment, we examine whether improvements in facial identity perception following high-frequency tRNS targeted at lateral occipitotemporal cortices are anatomically specific.

### Experiment 1

In Experiment 1, we examined the impact of active relative to sham high-frequency tRNS targeted at lateral occipitotemporal cortex on facial identity perception and facial trustworthiness perception. Based on prior studies, we predicted that active tRNS of lateral occipitotemporal cortices would modulate performance on facial identity perception, but not on trustworthiness perception (which may rely more on modulation from dorsomedial prefrontal cortex and inferior frontal gyrus—e.g., Bzodk et al. 2012).

## Experiment 1 Methods

### Participants

Thirty-six right-handed adults (23 females, M = 27 years, SD = 4 years) participated in this study for a small monetary reward. Participants were randomly assigned to active high-frequency tRNS (*N* = 18; mean age = 26.8 years, SD age = 3.5 years; 11 females) or sham group (*N* = 18; mean age = 27.2 years, SD age *=* 5.1 years; 12 females). The groups did not differ in terms of age (*P* = 0.793). All participants were healthy volunteers, without any known developmental or neurological disorders and no contraindications to tRNS. They were naive with respect to the experimental hypothesis and remained unaware of what type of stimulation they received until the end of the experiment. Participants provided written informed consent to take part in the experiment.

### Procedure

Prior to testing, all participants were provided with written information about the study and a description of the tRNS procedure. The associated safety/risk warnings were explained, and participants were asked to sign an informed consent form. This study received full ethical approval by the local ethics committee.

Participants received either 20 min of active high-frequency tRNS targeted at lateral occipitotemporal cortices or sham stimulation targeted at the same site. High-frequency tRNS was used in the current studies based on prior work, showing that this leads to greater increases in cortical excitability (Terney et al. 2008). The sites of stimulation were identified using the electroencephalography 10–20 system, with the center point of two 5 × 5 cm electrodes placed over P7/P8 electrode locations. Our selection of electrode site was based on prior work emphasizing the role of inferior temporal gyrus and middle temporal gyrus in face processing (Allison et al. 1994; Weiner and Grill-Spector 2013). Frameless stereotactic image guidance of scalp electrodes indicates that the P7/P8 electrodes are over these regions (Kim et al. 2007). Consistent with these findings, the face-sensitive N170 event related potential component is also greatest at P7/P8 (Bentin et al. 1996; Eimer 2000).

The stimulation was induced via a NeuroConn DC-Stimulator Plus. For active high-frequency tRNS, 1 mA current was delivered for 20 min with a 15-s fade-in and 15-s fade-out time. For the sham condition an identical set-up was used, but the stimulator was only turned on for 15 s. This condition evokes the sensation of being stimulated, but does not lead to a neurophysiological change that can influence performance. It has been shown that naive subjects cannot distinguish between sham and active tCS (Ambrus et al. 2010).

**Figure 1.**
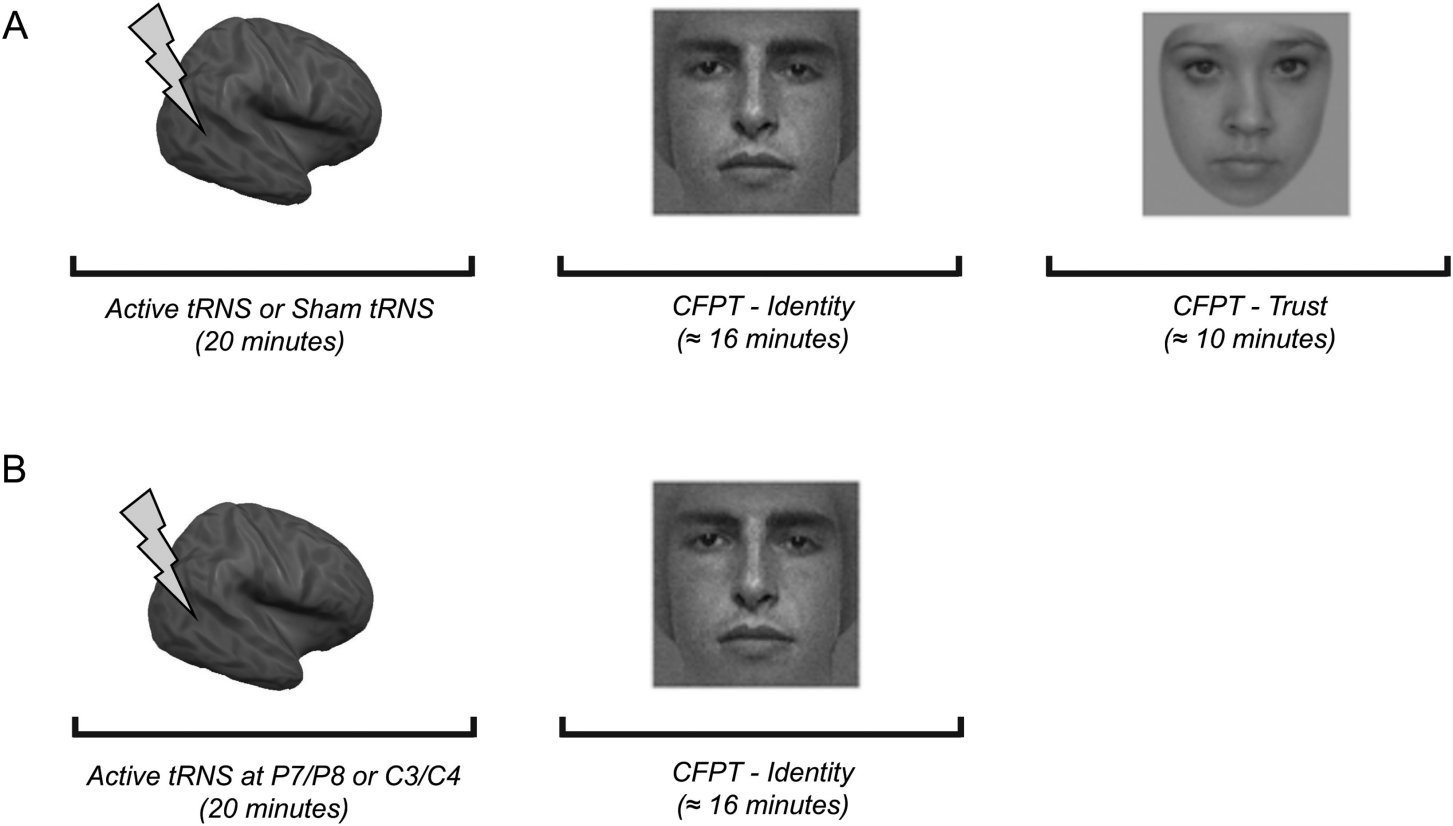
Experimental procedures in Experiments 1 and 2. (*A*) In Experiment 1, participants received 20 min of active or sham high-frequency tRNS targeted at lateral occipitotemporal cortices prior to completing 2 tasks (CFPT-Identity and CFPT-Trustworthiness) in a counterbalanced order. (*B*) In Experiment 2, participants received 20 min of active high-frequency tRNS targeted at lateral occipitotemporal or sensorimotor cortices prior to completing CFPT-Identity.

Following 20 min of offline stimulation or sham stimulation, participants completed 2 tasks in a counterbalanced order: Cambridge Face Perception Test-Identity (CFPT-Identity) and Cambridge Face Perception Test-Trustworthiness (CFPT-Trustworthiness; Fig. 1). The CFPT-Identity (previously called CFPT; Duchaine et al. 2007) assesses the ability to perceive differences between facial identities. Faces are presented simultaneously, therefore making memory demands minimal. During the task, participants were shown a target face (from a 3/4 viewpoint) and 6 faces (from a frontal view) morphed between the target and 6 other faces in varying proportions so that they varied systematically in their similarity to the target face. Participants were asked to sort the 6 faces by similarity to the target face and were given 60 s to do so. Eight sorts were used, and each sort was presented upright and inverted once in a fixed prerandom order. Performance on the CFPT-Identity is typically measured by an error score, which is calculated for each trial type. This is calculated by summing the deviations from the correct position for each face, with one error reflecting each position that a face must be moved to be in the correct location. For example, if a face was one position from the correct location, the error score was 1. If it was 3 positions away from the correct location, this was an error score of 3. Error scores on each trial type (upright vs. inverted) were summed to determine the total number of errors for each orientation. We then used this to calculate the percentage of correct responses. Chance performance for CFPT-Identity is 36%.

In the CFPT-Trustworthiness (Rezlescu et al. 2014), participants were asked to sort faces according to perceived trustworthiness. In each trial, participants were shown 6 faces from a frontal view with different levels of trustworthiness. Faces were not repeated in different trials. The correct sorting orders were determined based on average ratings obtained from 338 online participants (each average score included at least 48 data points). The 10 sorts were presented on the screen in a random order. Participants were required to sort them according to how trustworthy they appeared, from the face that looks least trustworthy on the left to the face that looks most trustworthy on the right. The time limit for each trial was set at 60 s. As per the CFPT-Identity, accuracy for CFPT-Trustworthiness was measured by calculating the percentage of correct responses, and chance performance is 36%.

## Experiment 1 Results and Discussion

A 3 (Task Type: CFPT-Identity upright, CFPT-Identity inverted, and CFPT-Trustworthiness) × 2 (Group: tRNS and sham) mixed ANOVA revealed a revealed a main effect of Task Type (*F*_2,68_ = 50.61, *P* < 0.001, $\eta_{P}^{2}$ = 0.598). This was due to participants performing better overall on CFPT-Identity upright relative to CFPT-Identity inverted and CFPT-Trustworthiness, and on CFPT-Trustworthiness relative to CFPT-Identity inverted (*P* < 0.005, Bonferroni-corrected in all cases). The main effect of group was also significant (*F*_1,34_ = 4.17, *P* = 0.049, $\eta_{P}^{2}$ = 0.109), which was due to participants in the stimulation group performing better overall compared with the sham group.

**Figure 2.**
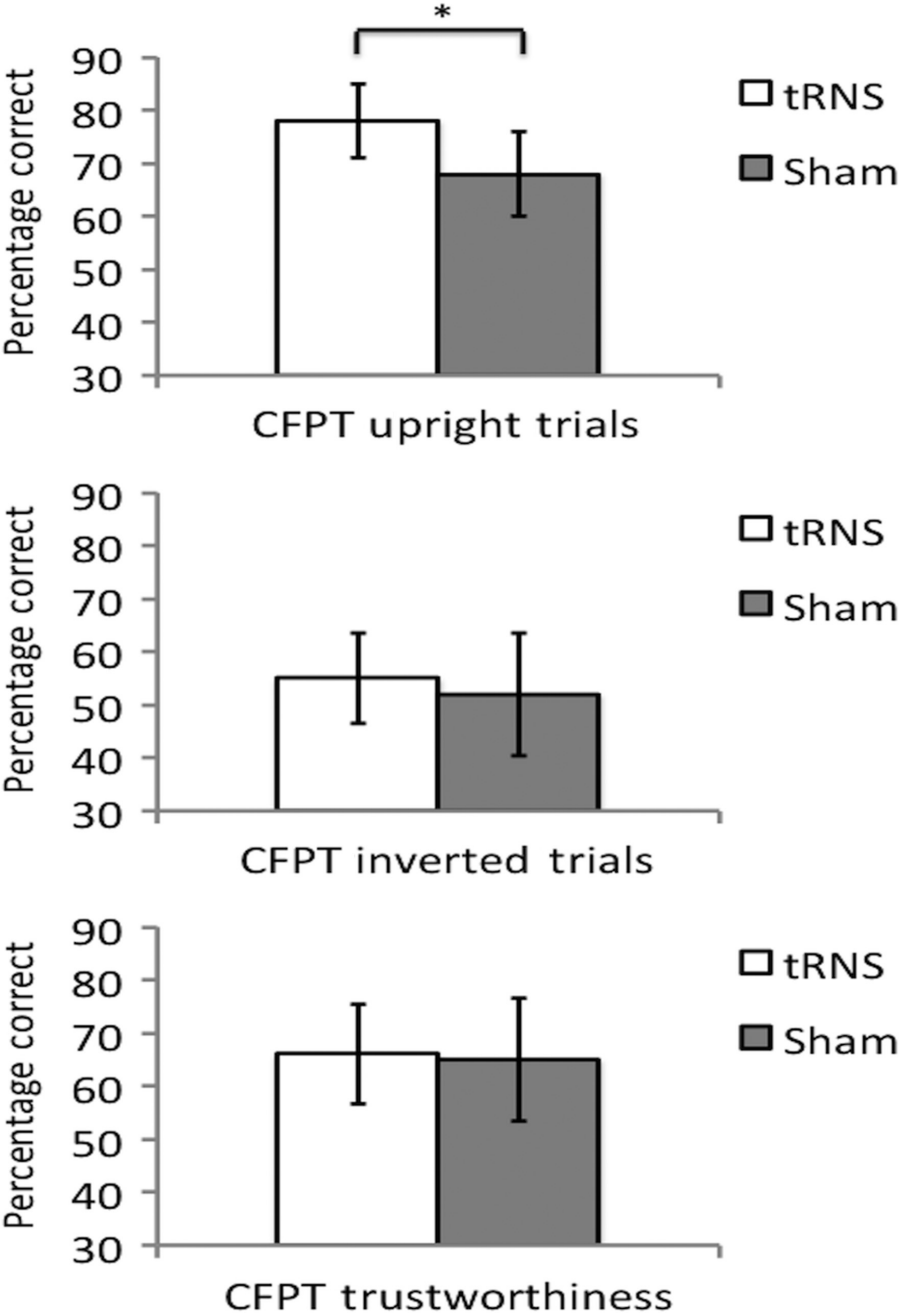
Experiment 1 Results. Performances following sham or active high-frequency tRNS targeted at lateral occipitotemporal cortices on CFPT-Identity and CFPT-Trustworthiness. Active high-frequency tRNS facilitated performance on CFPT-Identity upright trials, but not inverted trials. No significant differences were found between sham and active high-frequency tRNS on the CFPT-Trustworthiness. Chance performance is 36%. *P < 0.01.

Importantly, a significant interaction between Task Type and Group (*F*_2,68_ = 3.34, *P* = 0.041, $\eta_{P}^{2}$ = 0.089) was observed. Pairwise comparisons were conducted to compare the performances of each group. This revealed that the active tRNS group significantly outperformed the sham group on CFPT-Identity upright (*P* < 0.01), but not on CFPT-Identity inverted (*P* = 0.368) or CFPT-Trustworthiness (*P* = 0.841; Fig. 2). Therefore, high-frequency tRNS targeted at lateral occipitotemporal cortices facilitated upright facial identity perception in a task-specific manner.

### Experiment 2

While the findings from Experiment 1 demonstrated a task-specific facilitation in facial identity perception following high-frequency tRNS, the lack of active stimulation of a control brain region does not permit inference about anatomical specificity. To address this issue and to determine whether we could replicate our original findings, we ran a second experiment contrasting the effect of active high-frequency tRNS targeted at lateral occipitotemporal cortices and sensorimotor cortices. Based on the findings from Experiment 1, we predicted that high-frequency tRNS targeted at lateral occipitotemporal cortices would facilitate performance relative to sensorimotor cortex stimulation.

## Experiment 2 Methods

### Participants

Forty right-handed adults (22 females, M = 27.17 years, SD = 7.46 years) participated in this study for a small monetary reward. Participants were randomly assigned to the lateral occipitotemporal cortices (*N* = 20; mean age = 25.9 years, SD age = 8.25 years; 11 females) or sensorimotor cortices group (*N* = 20; mean age = 28.45 years, SD age = 6.54 years; 11 females). The groups did not significantly differ in age (*P* = 0.285). All participants were healthy volunteers, without any known developmental or neurological disorders and no contraindications to tRNS. They were naive with respect to the experimental hypothesis. Participants provided written informed consent to take part in the experiment.

### Procedure

Participants received either 20 min of tRNS targeted at P7/P8 or at C3/C4 prior to completing the CFPT-Identity. Lateral occipitotemporal cortices were again identified using the P7/P8 electrode sites (as per Experiment 1), whereas sensorimotor cortices were identified using C3/C4 scalp electrodes from the 10–20 EEG system. The stimulation was induced via two 5 × 5 cm surface electrodes placed on either site and was delivered by a NeuroConn DC-Stimulator Plus. 1 mA current was delivered for 20 min with a 15-s fade-in and 15-s fade-out time for each site. Following 20 min of offline stimulation, participants completed the CFPT-Identity (as per Experiment 1).

## Experiment 2 Results and Discussion

**Figure 3.**
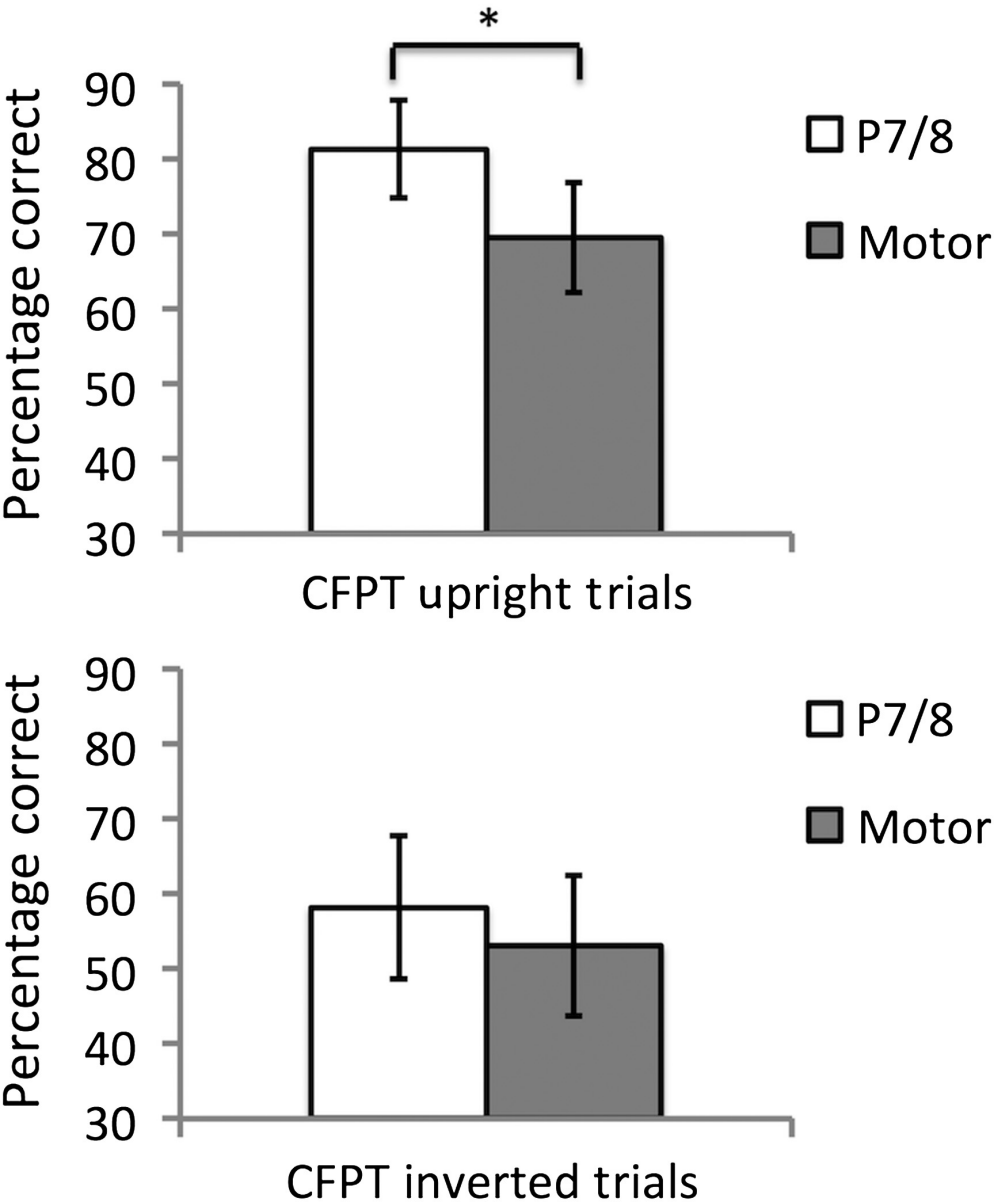
Experiment 2 Results. Performances following active high-frequency tRNS targeted at lateral occipitotemporal cortices or sensorimotor cortices on CFPT-Identity upright and inverted trials. The lateral occipitotemporal tRNS group outperformed the sensorimotor group. Analyses of trial-specific results revealed that the lateral occipitotemporal tRNS significantly facilitated performance on the CFPT-Identity upright trials relative to sensorimotor cortex stimulation, but not on CFPT inverted trials. *P < 0.001.

A 2 (Groups: occipitotemporal and sensorimotor) × 2 (Task Type: CFPT-Identity upright and CFPT-Identity inverted) ANOVA was conducted to compare differences in performance on each trial type following occipitotemporal or sensorimotor cortex stimulation. This revealed a significant main effect of group (*F*_1,38_ = 17.17, *P* < 0.001, $\eta_{P}^{2}$ = 0.311), with the occipitotemporal tRNS group outperforming the sensorimotor cortex stimulation group. There was also a main effect of task type (*F*_1,38_ = 143.49, *P* < 0.001, $\eta_{P}^{2}$ = 0.791), with participants performing better overall on upright relative to inverted CFPT-Identity (the face inversion effect—Yin 1969). There was also a borderline significant interaction between group and task type (*F*_1,38_ = 4.07, *P* = 0.051, $\eta_{P}^{2}$ = 0.097). Given this interaction and our prior predictions from Experiment 1, planned paired comparisons were conducted to compare performances between the groups on each task type. This revealed that, on CFPT-Identity upright, participants in the occipitotemporal group significantly outperformed participants in the sensorimotor cortex stimulation group [*t*_(38)_ = 5.38, *P* < 0.001; Fig. 3]. While there was a trend for better performance in the occipitotemporal group on CFPT-Identity inverted, no significant differences were found between the groups [*t*_(38)_ = 1.71, *P* = 0.095].

To further delineate the source of the difference between groups in Experiment 2, a secondary analysis was run comparing performances following active occipitotemporal or sensorimotor cortex high-frequency tRNS, relative to the sham stimulation condition from Experiment 1. This analysis was run to ensure that the pattern of data for experiment 2 held when comparing performances from active stimulation conditions to a condition in which no neurophysiological change due to high frequency tRNS took place. A 2 (Task Type) × 3 (Group) ANOVA revealed a main effect of Task (*F*_2,55_ =192.37, *P* < 0.001, $\eta_{P}^{2}$ = 0.778), which was due to participants performing better overall on upright relative to inverted trials (i.e., the face inversion effect). There was also a main effect of Group (*F*_2,55_ = 10.11, *P* < 0.001, $\eta_{P}^{2}$ = 0.269), which was due to the active occipitotemporal high-frequency tRNS group performing better overall relative to the active sensorimotor high-frequency tRNS group and sham group (*P* < 0.005 in each case). Importantly, there was also a significant Group × Task interaction (*F*_2,55_ = 3.28, *P* = 0.045, $\eta_{P}^{2}$ = 0.107). Paired comparisons revealed that this was due to participants in the active high-frequency tRNS occipitotemporal group showing superior performance relative to the active sensorimotor high-frequency tRNS group (*P* < 0.001) and sham tRNS group (*P* < 0.001) on upright, but not on inverted trials (sensorimotor tRNS group comparison—*P* = 0.095; sham tRNS group comparison—*P* = 0.102). No significant differences were found between the sensorimotor high-frequency tRNS group and sham tRNS group on upright (*P* = 0.546) or inverted (*P* = 0.858) trials.

Collectively, these findings are consistent with our findings from Experiment 1 by showing that tRNS targeted at occipitotemporal cortices facilitates facial identity perception abilities, although in Experiment 2 there was also a trend for improvement on inverted face perception. The findings from Experiment 2 highlight a level of anatomical specificity for the tRNS effect by showing that high-frequency tRNS targeted at occipitotemporal cortices significantly facilitated facial identity perception abilities relative to stimulation at the sensorimotor cortices. The trend with inverted faces is consistent with the idea that inverted face perception involves some of the same mechanisms as upright face processing (Freiwald et al. 2009; Pitcher et al. 2011; Susilo et al. 2013) and the spatial specificity of high-frequency tRNS making targeting neural regions involved solely in upright face perception unlikely.

## General Discussion

The aim of this study was to examine the potential for using high-frequency tRNS as a tool to enhance face perception abilities. Based on prior neuroimaging studies of face processing [e.g., see Weiner and Grill-Spector (2013)] and tRNS work in other domains (e.g., numerical cognition; Cappelletti et al. 2013; Snowball et al. 2013), we anticipated that a single session of active high-frequency tRNS targeted at lateral occipitotemporal cortices would enhance facial identity perception abilities. We found this to be the case across 2 experiments. In Experiment 1, we observed that active relative to sham tRNS targeted at lateral occipitotemporal cortices enhanced performance on perception of upright facial identity, but not of inverted facial identity or perception of facial trustworthiness. In Experiment 2, we replicated this result and found evidence for anatomical specificity by observing that active high-frequency tRNS targeted at lateral occipitotemporal cortices enhanced performance on perception of facial identity relative to sensorimotor cortices high-frequency tRNS. Collectively, these findings imply that a single session of high-frequency tRNS targeted at bilateral occipitotemporal cortices can improve facial identity perception abilities in a task (Experiment 1) and site-specific (Experiment 2) manner.

The results demonstrate the potential utility of high-frequency tRNS as a tool to improve facial identity perception. In doing so, the findings highlight the potential for high-frequency tRNS to be employed as a tool to improve face perception in typical adults and suggest that tRNS may be an effective intervention strategy in conditions in which face recognition abilities are impaired. However, given potential differences in levels of cortical excitation between typical and atypical groups, it will be important that any future attempts to extend these findings to atypical groups carefully consider additional factors influencing baseline levels of cortical excitation that may influence the efficacy and direction of stimulation effects in groups with atypical face processing (also see Krause and Cohen Kadosh 2014).

Furthermore, it is important to consider the mechanisms that mediate the improvements in face perception observed here. Although the current findings provide evidence of a task-specific modulation of facial identity perception abilities but not of trustworthiness perception, some caution is needed when considering the mechanisms that drive this improvement. The format of CFPT-Identity and CFPT-Trustworthiness are subtly different because in the identity task participants are required to make fine-grained visual judgements regarding how well each image matches a target face in a different viewpoint, whereas in the trustworthiness task there is no target face and attention to particular features may not be critical. With this in mind, it could be argued that the improvement found in CFPT-Identity upright is related to an improvement in the ability to make a fine-grained visual discrimination between one set of images and a target stimulus. While the findings from Experiment 1 would argue against this account because no improvement was found on CFPT-Identity-inverted trials, the findings of a trend for improvement on inverted trials in Experiment 2 suggest that some caution is required in assuming that enhancement of domain-specific mechanisms fully accounts for the improvements observed across the studies.

In this context, it is also interesting to discuss the absence of a significant improvement on CFPT-Identity-inverted trials in the 2 studies reported here. The extent to which upright versus inverted face processing relies on similar neurocognitive mechanisms is equivocal. With this in mind, there are at least 2 ways to interpret the lack of effect on inverted faces. First, it could be argued that our data fit with prior work, suggesting that upright and inverted face perception are dependent on qualitatively different neurocognitive processes (e.g., Tanaka and Farah 1993; Moscovitch et al. 1997; Yovel and Kanwisher 2004; McKone and Yovel 2009; Pitcher et al. 2011). Alternatively, the lack of an effect on inverted trials may reflect a quantitative difference in the extent to which upright and inverted faces activate common neurocognitive mechanisms. This account would be consistent with suggestions that some common neurocognitive processes are recruited for upright and inverted face processing (e.g., Freiwald et al. 2009; Pitcher et al. 2011; Susilo et al. 2013), but that these processes are facilitated when faces are presented upright (e.g., Sekuler et al. 2004; Gold et al. 2012). Given that one means through which tRNS is believed to influence perceptual performance is by amplifying neural signals (e.g., through principles of stochastic resonance—Moss et al. 2004), a stronger signal within a brain area at baseline may lead to performance on upright face perception being more likely to show behavioral gain from stimulation than inverted face perception. It is therefore feasible that a more powerful stimulation method may lead to similar patterns of improvement on inverted trials. This is an important question for future investigation.

More broadly, it will also be interesting to examine the interaction between pairing the stimulation parameters used here with face training paradigms, especially in groups with atypical face processing abilities. In other domains, combining brain stimulation with cognitive training has been shown to significantly improve the benefits of training or stimulation alone, and to be maintained over time (e.g., Cohen Kadosh et al. 2010; Cappelletti et al. 2013; Snowball et al. 2013). The potential to enhance face processing via training paradigms has a long history, with evidence of effective interventions only recently emerging [e.g., see DeGutis et al. (2014) for review]. Attempting to improve the efficacy of intervention strategies designed to aid face perception abilities by combining these with brain stimulation will be an important future direction for this work.

In summary, the present findings reveal that a single session of high-frequency tRNS targeted at lateral occipitotemporal cortices can enhance facial identity perception. This improvement was both site- and task-specific, and suggests that high-frequency tRNS has the potential to be a useful tool to improve face perception abilities. It will be important for future work to extend on the single-session improvements observed here by combining brain stimulation with face training paradigms and assessing the extent to which improvements generalize across different face processing tasks. The insights gained from the current and future directions will hopefully aid in the development of novel intervention strategies to improve face perception abilities in typical and atypical groups.

## Funding

This work was supported by grants from the British Academy (SG111874) and ESRC (ES/K00882X/1) awarded to M.J.B. A.R. was also supported by a Wellcome Trust Biomedical Vacation Scholarship (WT102340MA). Funding to pay the Open Access publication charges for this article was provided by RCUK (ESRC) Open Access Block Grant (Goldsmiths).
